# Using Digital Media to Improve Dementia Care in India: Protocol for a Randomized Controlled Trial

**DOI:** 10.2196/38456

**Published:** 2022-06-02

**Authors:** Bianca Brijnath, Upasana Baruah, Josefine Antoniades, Mathew Varghese, Claudia Cooper, Briony Dow, Mike Kent, Santosh Loganathan

**Affiliations:** 1 National Ageing Research Institute Parkville Australia; 2 Institute of Human Behaviour & Allied Sciences Delhi India; 3 National Institute of Mental Health and Neuro Sciences Bengaluru India; 4 University College London London United Kingdom; 5 Curtin University Perth Australia

**Keywords:** dementia, care, India, digital health, film

## Abstract

**Background:**

India is undergoing a demographic transition characterized by population aging and is witnessing a high dementia rate. Although nearly 7 million people live with dementia in India, dementia awareness is poor, and current resources addressing dementia care are basic and often incomplete, duplicated, or conflicting. To address this gap, this study aims to use digital media, which has had a massive technological uptake in India, to improve dementia care in India.

**Objective:**

The objective of this paper is to describe an intervention study design that examines the feasibility and acceptability of Moving Pictures India, a digital media resource to improve dementia care in India.

**Methods:**

This study employs a mixed methods design and is divided into 4 phases: (1) video interviews with Indian caregivers and health professionals; (2) coproduction of resources; (3) pilot randomized controlled trial (RCT); and (4) dissemination and analytics. The pilot RCT will follow an experimental parallel group design with 2 arms aiming to assess the impact, feasibility, and acceptability of the developed resources. The primary outcome measures for the pilot RCT will be feasibility and acceptability, while the secondary outcome measures will be caregiver burden, mood, and quality of life.

**Results:**

This study received funding from the Alzheimer’s Association in the United States in July 2021. In 2023, we will enroll 60 dementia caregivers (40 caregivers in the intervention arm and 20 in the control) for the pilot RCT. The study has been approved by the National Institute of Mental Health and Neuro Sciences Ethics Committee (26th IEC (BEH.SC.DIV.)/2020-21 dated November 11, 2020); the Health Ministry's Screening Committee, India (proposal ID 2020-10137); the Curtin University Human Research Ethics Committee (approval number HRE2020-0735); and the NARI Research Governance Office (site-specific approval dated March 17, 2021).

**Conclusions:**

This protocol is designed to deliver unique, coproduced, and evidence-based media resources to support caregivers of persons with dementia in India and other countries aiming to utilize digital media for dementia care. If the intervention is found feasible and acceptable, postpiloting analytics and qualitative feedback will be used to develop an implementation trial to evaluate the effectiveness of the potential low-risk high-benefit intervention in practice.

**Trial Registration:**

Clinical Trials Registry-India CTRI/2021/01/030403; http://ctri.nic.in/Clinicaltrials/pmaindet2.php?trialid=50794

**International Registered Report Identifier (IRRID):**

DERR1-10.2196/38456

## Introduction

India, like the rest of the world, is experiencing population aging, and this demographic transition will have a significant impact on age-related conditions such as dementia [1]. With nearly 7 million people affected [2], India has the fastest growing dementia rate worldwide and will soon host one of the largest populations of people with dementia. A large proportion of people with dementia are cared for by their immediate and extended families [3]. The role of family is paramount in dementia care in India and is rooted in the traditional concept of “seva,” which emphasizes service, honor, and respect for older people and a social expectation that they will be cared for by their children [4].

Despite affecting millions in India, the symptoms of dementia are often viewed as a normal part of aging, and there is a lack of awareness about the symptoms, treatment, and challenges associated with caring for a person with dementia [5]. Some family caregivers have no basic knowledge of dementia; many, despite receiving a dementia diagnosis, do not understand the implications of the diagnosis for themselves or their relatives with dementia [1,6]. Limited understanding can result in the person with dementia not receiving the necessary support and treatment to address their needs and improve their well-being, and it can also lead caregivers to feel publicly stigmatized, ashamed, or distressed [5]. Concomitant with a lack of care infrastructure and scarcity of resources, such as respite- and long-term care facilities, is the fact that family caregivers are often very isolated and without support [7]. In these circumstances, caregivers often experience increased burden, a decline in their mental and physical health, and a loss of income and productivity because of caregiving [8-11].

To ameliorate these burdens, greater focus on imparting knowledge and skills to empower caregivers for persons with dementia is crucial [5]. As many family caregivers enter their new role unprepared, they need information about what dementia care involves, care pathways, social and clinical care, sources of help, and other services available [12]. Provision of culturally salient dementia care resources, during diagnosis and postdiagnosis, may help caregivers gain perspective about their family member’s behaviors. Such resources would also help families understand dementia as a medical condition rather than attributing symptoms to moral deficits in the person themselves or in the care provided by their caregivers.

Technological innovations, especially digital media, can help improve dementia care. Digital media, particularly smartphones, can help caregivers access information online, monitor their own physiological and biometric data along with that of the person with dementia, and set reminders for medications and other therapies [13,14]. According to a report by the India Cellular & Electronics Association, nearly 830 million people in India are smartphone users, thus making smartphones and digital media crucial dissemination platforms for content creators and service providers [15].

In Australia, an innovative multimedia project called Moving Pictures is producing short films on dementia care, which are coproduced with people from culturally and linguistically diverse backgrounds [16]. The project is a collaboration between the National Ageing Research Institute (NARI) and Curtin University, and the films produced are based on the stories and lived experiences of family caregivers of people living with dementia as well as the expert views of key service providers. Taking into context the need for dementia care resources and the current technological uptake in India, authors from India, Australia, and UK have collaborated to develop Moving Pictures India to improve dementia care in India using digital media. The aim of Moving Pictures India is to coproduce 9 short films on common issues in dementia care with caregivers and stakeholders and to pilot the resources developed to determine whether the findings would support a large implementation trial. There are 4 main objectives of the study: (1) to video interview Indian caregivers and health professionals about cultural understandings of dementia, social and clinical care, sources of help, and care pathways; (2) coproduce 9 short films on dementia care with caregivers and stakeholders; (3) pilot the resources with caregivers to assess feasibility and acceptability using a randomized controlled trial (RCT) design; and (4) disseminate the films online for free and use analytics and qualitative feedback to develop an implementation trial. This paper describes the protocol of the study.

## Methods

### Methodological Approach

The methodological approach for the study follows the United Kingdom Medical Research Council guidelines for the development and evaluation of complex interventions [17]. The process of designing and evaluating complex health interventions includes development, feasibility and piloting, evaluation, reporting, and implementation. Mobilizing this methodology, this study uses robust mixed methods including video interviews, stakeholder consultation, community forums, a quasi-experimental trial, and big data analytics. Owing to its mixed methods design, this study is divided into 4 different phases based on the main objectives. The methods of each phase including the research design are described as follows.

#### Phase 1. Video Interviews: Intervention Development

In the first year of the study, video interviews with family caregivers and health professionals will be recorded to create the films for mobile devices targeting family caregivers and the Indian public. Family caregiver interviews will focus on initial perceptions of dementia symptoms, at what point medical intervention was sought and why, examples of successful collaboration between caregivers and services, and how the dementia care pathway unfolds for families. Health professional interviews will focus on working with doctors; going to the hospital; pain; eating, nutrition, and dental care; hygiene and incontinence; and later stages and palliative care. The topics for discussion in the interviews are based on patient and public involvement and clinician consultation. 

##### Inclusion Criteria

We will recruit family caregivers who are currently caring for a family member with dementia for at least 6 months. Health professionals include medical specialists (eg, geriatricians, old age psychiatrists), nurses, allied health care workers (eg, physiotherapists, social workers), or related professionals (eg, care coordinators, direct care workers) involved in providing health and community services to people living with dementia. We will include participants who speak either English, Hindi, or Kannada, have the capacity to consent, and are willing to participate in a video interview, snippets of which will be included in the final Moving Pictures India films and resources.

##### Recruitment and Sampling

The aim is to purposively recruit 25 family caregivers speaking either of the 3 aforementioned languages and representing different genders and socioeconomic classes, along with 25 health professionals who satisfy the inclusion criteria. The sample size was determined based on the research team’s extensive experience of sampling to ensure sufficient diversity. Participants will be invited to participate in a 30- to 60-minute video interview conducted in English, Hindi, or Kannada. Purposive and snowball sampling will be employed for recruitment. Data will be collected in Bengaluru, India.

Upon confirmation of interest, the researcher will forward a copy of the participant information and consent form (PICF) either by email or regular mail with a prepaid envelope. Contact will be made a few days later to gauge interest, answer any relevant questions, and arrange the interview. If the consent form is not received by the research team prior to the interview, it will be completed before starting the interview. All participants will be provided with a verbal overview of the study, time to read the PICF (if not done earlier), and an opportunity to seek clarification about any study-related matter from the researcher prior to the interview.

##### Data Analysis

Data will be translated, transcribed into English, and thematically analyzed [18-20] by the research team using NVivo version 12 or the latest version of the software. Deductive analysis will be used, informed by the cross-cultural care literature, and will focus specifically on how concepts such as aging (eg, “budhapan”), dementia (eg, “yadarsht khona” or losing memory), dementia symptoms (eg, “bak bak” or useless, repetitive talk), and care (eg, “seva”) are understood cross-culturally and in light of India’s rapid modernization and reduced availability of adult children to provide familial care. Digital resources will be cut from the recordings.

#### Phase 2. Coproduction of Films and Resources: Intervention Development

In the second year, the data from the first phase will be storyboarded to form the basis of 9 films and animations. The themes for the storyboards are based on common issues in dementia care arrived at through the researchers’ prior work [4,16] and will focus on the following themes: (1) signs of dementia; (2) care pathways; (3) self-help for caregivers; (4) recognizing and responding to unmet needs; (5) working with doctors and going to hospital; (6) pain; (7) eating, nutrition, and dental care; (8) hygiene and incontinence; and (9) later stages and palliative care. Storyboards will be internally reviewed for cultural appropriateness followed by realist and critical evaluation at a stakeholder workshop in Bengaluru using the nominal group technique. The nominal group technique is designed to democratically elicit ideas and is widely used in health and education research. It involves group members first working alone, then sharing ideas and undertaking group discussions, and finally, voting and ranking [21]. Stakeholders at the workshop will include the investigator team, film editor, dementia services, clinicians, researchers, and artists. Following stakeholder approval, the storyboards will be converted to Hindi/Kannada films (by the film editor) and animations (by the designer).

**Figure 1:**
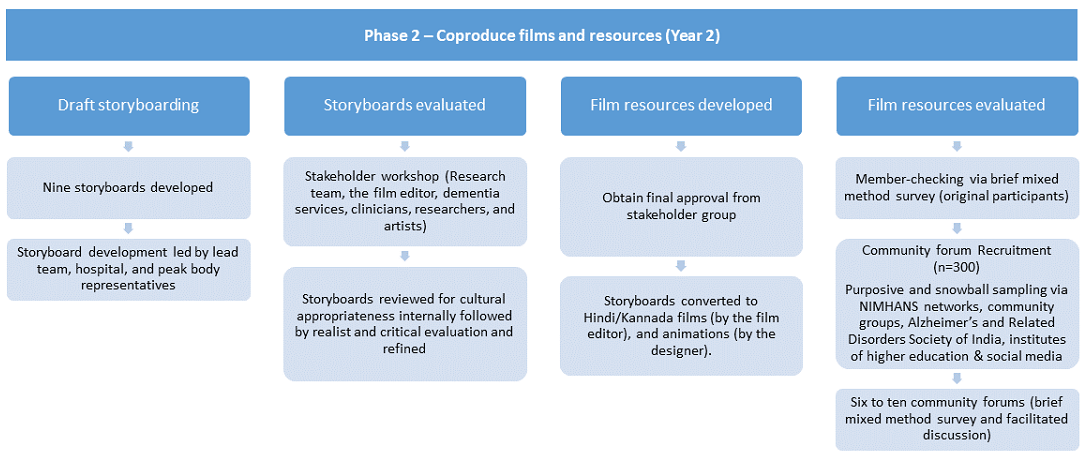
Study protocol flowchart of phase 2: Coproduction of films and resources. NIMHANS: National Institute of Mental Health and Neuro Sciences.

To evaluate the appropriateness of the films and animations, they will be member-checked with the original interview participants as well as with community members to ensure believability and trustworthiness [22]. Interview participants and community members will complete a survey (eg, whether expectations were met, level of enjoyment and interaction, key learnings, etc) in English, Hindi, and/or Kannada via telephone or online. Film and animation screenings with community members will be completed using a workshop format, and feedback will be used to improve the resources. A flowchart of this phase is illustrated in Figure 1.

##### Inclusion Criteria

Members of stakeholder groups and the community members must be 18 years of age or over, have a smartphone, have the capacity to provide consent, and be literate in either English, Hindi, or Kannada.

##### Recruitment and Sampling

Family caregivers and health professionals who participated in phase 1 of the study will be asked to provide feedback on resources once they are completed. Community members (n=~300) speaking either of the 3 aforementioned languages, representing different genders and socioeconomic classes, and satisfying the inclusion criteria will be recruited in Bengaluru to test the films and resources. Bengaluru is one of the most multicultural and ethnically diverse cities in India and has a large migrant population due to its status as the information technology hub of the country [23,24]. Recruitment will be facilitated via invitations/advertisement (through email, hard copies, social media posts, newsletters, and public notices) to local senior citizens’ groups, social groups, Alzheimer’s and Related Disorders Society of India Bengaluru Chapter, community and health services, and institutes of higher education. Upon request, a member of the research team will give a talk about the study to groups of interested individuals. Participants will be invited to participate in one of 6-10 workshops (1-2 hours) in which films and resources will be screened and evaluated.

#### Phase 3. Pilot RCT: Intervention Assessment

**Figure 2:**
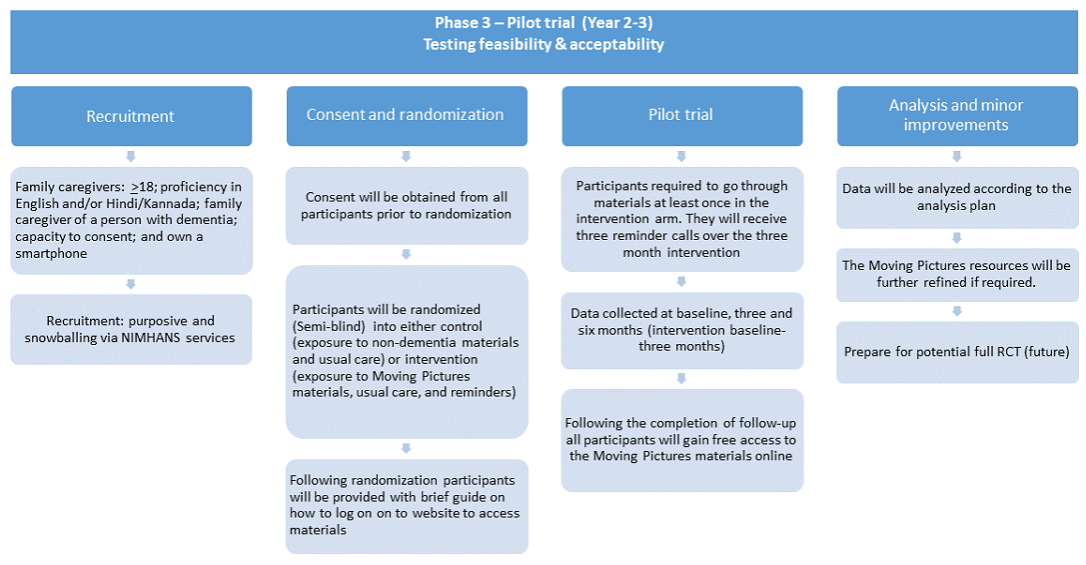
Study protocol flowchart of phase 3: pilot randomized controlled trial. NIMHANS: National Institute of Mental Health and Neuro Sciences; RCT: randomized controlled trial.

To evaluate the impact of the resources produced, a pilot RCT will be conducted in the second and third year of the study (Figure 2). The pilot RCT will follow an experimental parallel group design with two arms aiming to assess the impact, feasibility, and acceptability of the resources of Moving Pictures India. The aims of the pilot RCT will be: (1) to test the feasibility of a full RCT with feasibility prespecified as >70% of participants expressing interest, consenting to participate, and >80% participation in follow up; (2) to test acceptability, prespecified as >70% of participants rating the intervention “very acceptable.”

##### Inclusion and Exclusion Criteria

Participant inclusion criteria are aged 18 years and up, proficient in English and/or Hindi/Kannada, self-reported family caregiver of a person with dementia who has been involved in the caregiving process for at least 6 months, capacity to consent, and own a smartphone. Participants involved in the development phase will be excluded.

##### Recruitment and Sampling

Participants will be recruited in Bengaluru, India. Purposive and snowball sampling will be employed for recruitment. Family caregivers who accompany people with dementia to the Geriatric Clinic and Services Unit at the National Institute of Mental Health and Neuro Sciences (NIMHANS) will be recruited. Furthermore, advertisements will be posted in public spaces in the NIMHANS facility and distributed in hard or soft copy by the research team to relevant clients, through relevant NIMHANS services and other support and social groups. The materials will contain contact details for the NIMHANS-based research team (email/phone). Potential participants will be able to contact the research team to express their interest in the study. The researcher will describe the study procedures, and if the participant agrees to the study, the researcher will confirm the participant’s eligibility. Alternatively, if potential participants learn about the study from a NIMHANS service provider, the latter will provide the contact details of the research team or, with verbal consent from the interested individual, forward their details to the research team, who will make the initial contact to provide the necessary study information. Upon confirmation of interest, the researcher will forward a copy of the PICF either by email or regular mail with a prepaid envelope, or it can be obtained from the research team located at NIMHANS upon request. Contact through phone or email will be made a few days later to confirm interest, answer any questions, and arrange for a brief meeting to sign the consent form. Signed consent can also be provided by mail or electronically.

##### Randomization and Blinding

Following consent, participants will be randomized into either an intervention arm that comprises usual clinical care plus access and use of the digital resources or a control arm comprising usual clinical care and access to nondementia resources (eg, a pamphlet on healthy living). Randomization will be done using computer-generated random number sequences, and the process will be overseen by a researcher not involved in the study. After allocation to groups, participants will be able to choose to receive specific instructions on how to access materials relevant to the condition (a brief instructional booklet) they have been allocated to either electronically, by regular mail, or in person. As a booster, participants in the intervention arm will also receive a monthly phone call from the research associate to help sustain intervention engagement over the long term, which is a risk to any digital trial [25]. Caregivers will not be told whether they are in the intervention or the control group to reduce potential bias. However, this blinding is not expected to be of any disadvantage, as all participants will have free and unlimited access to the Moving Pictures India materials after the pilot RCT.

##### Outcome Measures

The primary outcome measures for the pilot RCT will be feasibility and acceptability, while the secondary outcome measures for the pilot RCT will be (1) caregiver burden, as measured by the Zarit Burden Interview [26]; (2) caregiver mood, as measured by the Center for Epidemiological Studies Depression 10-item scale [27]; and (3) caregiver quality of life, as measured by the World Health Organization Quality of Life Scale [28].

**Figure 3:**
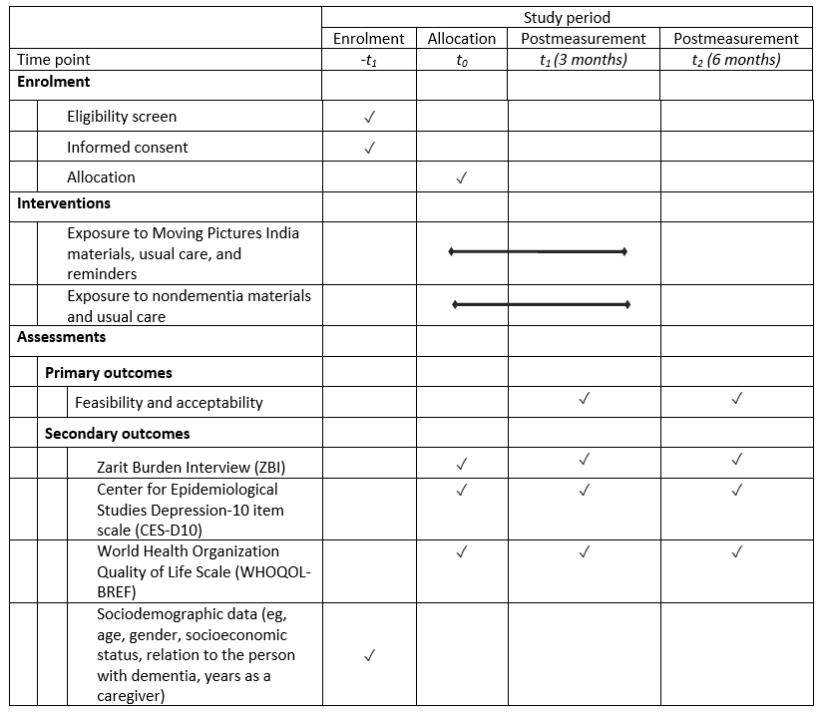
SPIRIT (Standard Protocol Items: Recommendations for Interventional Trials) flowchart of the schedule of enrollment, interventions, and assessments for the phase 3 pilot randomized controlled trial.

Validated, translated versions of the Zarit Burden Interview and World Health Organization Quality of Life Scale measures are already available in English, Hindi, and several other Indian languages. All the secondary outcome measures will be self-rated and measured at baseline, 3 months, and 6 months via regular mail, telephone, or online. Sociodemographic data (eg, age, gender, socioeconomic status, relation to the person with dementia, years as a caregiver) will also be collected. The schedule of enrolment, interventions, and assessments for the pilot RCT is presented in Figure 3.

##### Statistical Analysis

To estimate parameters of feasibility and acceptability with sufficient precision, which will help inform the future implementation trial, 40 participants from the intervention arm and 20 from the control arm are expected. The expected values for the proportion of family caregivers rating the intervention as “very acceptable” are 70% (95% CI 59%-87%) or 70% (95% CI 62%-85%) for participants for whom outcomes are completed at the 6-month follow-up. Achieving these expected values will be a priori criteria for progression to an implementation trial without modification of the intervention.

Repeat measures ANOVA will examine changes over time. The proportions of eligible family caregivers approached who agreed to take part in the study will be reported with a 95% CI, and any reasons for refusal will be summarized. Characteristics of people with dementia and their family caregivers that are included in the study will be summarized using means (with SD), medians (with interquartile ranges), counts, and proportions, as appropriate. Scores measured pre- and postintervention will be summarized at each time point (eg, using means and SD) and as differences between the randomization groups with 95% CI. Completeness of these scores will also be described. We will estimate parameters to help inform development of the implementation trial, particularly, the proportion of family caregivers using the intervention and for whom outcomes were completed at the 6-month follow-up.

##### Postpilot Revision

If required, specific changes based on the research team’s postpilot experience and participant feedback will be incorporated into the intervention before dissemination of the resources.

#### Phase 4. Dissemination and Analytics: Intervention Dissemination

**Figure 4:**
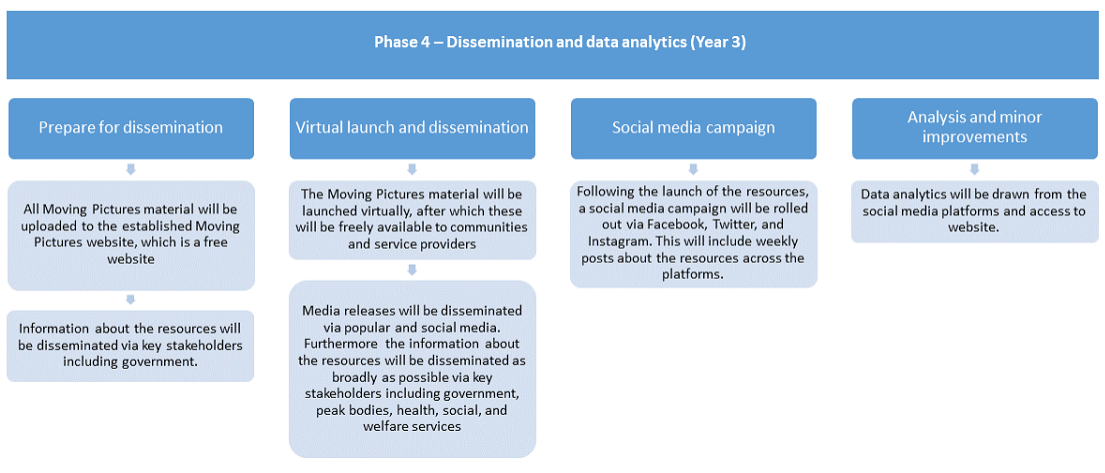
Study protocol flowchart of phase 4: dissemination and analytics.

Following postpilot minor revisions (if any), all the resources developed in the study will be disseminated via the mobile-optimized Moving Pictures website. Key stakeholders (eg, the Indian Ministries of Health and Social Justice, state and local government health and welfare offices, Alzheimer’s and Related Disorders Society of India, Alzheimer’s International; Alzheimer’s Association in the United States, community groups, health services, and media) and consumers (caregivers, people with dementia, and the general public) will receive a media release with links to the website. Social media campaigns will also be run at regular intervals on Facebook, YouTube, WhatsApp, and Twitter. To evaluate the reach and uptake of these digital resources, analytics data will examine website traffic and viewing trends over 6 months. The feedback in the comment sections will be reviewed and visits to the website will be counted. A flowchart of this phase is illustrated in Figure 4.

### Ethics Approval

The procedures outlined in this study are in accordance with the Declaration of Helsinki, and this study has been approved by the NIMHANS Ethics Committee (26th IEC (BEH.SC.DIV.)/2020-21 dated November 11, 2020); the Health Ministry's Screening Committee, India (proposal ID 2020-10137); the Curtin University Human Research Ethics Committee (approval number HRE2020-0735); and the NARI Research Governance Office (site-specific approval dated March 17, 2021).

### Data Storage and Transfer

As the data are being collected in India, data will be stored securely onsite; specifically, hard copy data will be stored in a locked filing cabinet in the office of the investigators. Signed documents will be converted to electronic files, which will then be password protected and stored on the secured NIMHANS network. When in the field, data will be stored on the research staff member’s laptop, which is password protected and backed up to an external hard drive that will also be password protected if required. All data (hard and soft copies) will be stored securely stored and destroyed 7 years after publication. Electronic copies of all data will be securely shared with the Australian research team. During data analysis and write-up, the data will be stored on a secured server in the password-protected folder only accessible by the Moving Pictures research team. Upon completion, all data will be securely transferred and stored under secure conditions at Curtin University and NARI, Australia, for 7 years after the research is published and then securely destroyed.

### Project Dissemination

Data will be published in deidentified form in academic and other publications (book chapters, conference presentations, media releases, social media posts, etc) under pseudonyms or participant IDs. For example, the participant might be described in the following manner: Asha Chand (pseudonym), female, age, caregiver for mother. Data used in films and resources may be identifiable, as we will be using the video data from the interviews in the resources. Caregivers will be assigned pseudonyms, and health providers will be using their own names and affiliations as specified in the PICFs. The films will be accessible via the Moving Pictures website to anyone in the community, and efforts will be made to disseminate the resources as broadly as possible. There are no plans to make the dataset publicly available after deidentification.

## Results

This study was funded by the Alzheimer’s Association. It brings together complementary expertise across multiple disciplines such as anthropology, psychology, psychiatry, geriatrics, film and media studies, disability studies, and social work.

Postdissemination analytics and qualitative feedback will be used to develop an implementation trial; explore how different strategies for dissemination and integrating the resources in local service provisions impact relevant caregiver variables like caregiver burden, mood, and quality of life; and evaluate the effects of the approaches to improve the uptake and utilization of the intervention. From July to December 2023, we will enroll 60 dementia caregivers (40 caregivers in the intervention group and 20 in the control) for the pilot RCT and follow them up for 6 months. This study is expected to conclude in June 2024.

## Discussion

### Overview

Although dementia has been recognized as a global public health priority, a huge gap between dementia care needs and resources still exists, especially in low- and middle-income countries. There is a clear need for the development of dementia literacy and care resources that emphasize identifying dementia through awareness, offer interventions to manage the symptoms of dementia, and promote well-being of both the caregiver and the person with dementia. In this paper, we have outlined the design of an intervention study that involves the development of a digital media resource to improve dementia care in India and determine the feasibility and acceptability of the developed resource using an RCT design. This study will be one of the first to deliver coproduced, evidence-based media resources to support caregivers of people with dementia in the Indian context.

### Comparison With Prior Work

The flexible, nonlinear approach of the UK Medical Research Council framework that guides the methodology of this study has previously been used in the design processes for the development of new technology-based interventions across disciplines [29-31]. This protocol emphasizes the importance of all the stages involved in developing and evaluating complex interventions, such as Moving Pictures India, and provides equal focus on development and piloting work as it does to the evaluation and proper consideration of the practical issues of implementation.

Through the process of coproducing films, we will gain an understanding of how dementia is perceived cross-culturally. This is an important aspect for dementia literacy and care, as dementia in India is typically viewed as a natural part of aging with cultural explanations used to describe dementia such as “chinnan” (childishness) [32]. The outcomes defined for the study, as well as the instruments to measure the outcomes, have been used in earlier studies with caregivers of people with dementia [29,33-36].

### Limitations

Although there are no foreseeable risks associated with participation in this study, there are some factors that may hinder recruitment and participant retention. Digital media is not a common support intervention for caregivers of persons with dementia in India, and acceptance of the program, especially by older caregivers such as spouses, could hamper recruitment. Lack of time to take part in the study due to care responsibilities could also be a factor in retention and recruitment. Nonadherence in eHealth interventions has been reported to be high in earlier studies [37,38]. Another limitation could be that only motivated and active caregivers who are internet or media savvy will take part in the study, which could possibly limit the generalizability of the results and acceptance of the program for the anticipated larger trial. Notwithstanding the limitations, the protocol includes potentially useful approaches for implementation, the most significant of which is the involvement of stakeholders and the utilization of a multifaceted approach involving a mixture of interactive workshops, feedback, reminders, and local consensus processes in the design of the research to ensure relevance [39,40].

### Conclusions

This research protocol directly addresses a looming public health crisis in India by promoting dementia awareness and developing resources to support caregivers in delivering high-quality care at home. It offers solutions that are culturally specific, scalable, and based on knowledge of local resources and burden of disease [41]. Thus, it has the potential to improve the lives of millions of people in India. The design selected leverages India’s significant online presence, and resources will be available to those who have difficulty accessing face-to-face information and support on account of being too busy, having difficulty traveling, and/or living in rural areas. Importantly, the lessons learned from this study are highly transferable. India also has a diaspora of 30 million people, the largest in the world [42]. Concerns regarding suboptimal dementia care in Indian communities have been expressed by caregivers and key service providers in the Moving Pictures Australia study [16]. Similar concerns have also been echoed from studies in the United Kingdom and Canada [43,44]. With the integrated insights from Moving Pictures Australia and Moving Pictures India, this protocol will yield an intervention that will be transferrable to multiple diaspora communities in low-, middle-, and high-income countries and will explore how different strategies for dissemination and integrating resources in local service provisions could impact caregiver burden, mood, and quality of life.

## Appendix

Multimedia Appendix 1 Blinded peer-review assessment scores and comments from the Alzheimer's Association Research Grant Review Committee - Alzheimer's Association Research Grant (AARG).

## Abbreviations

NARI: National Ageing Research Institute
NIMHANS: National Institute of Mental Health and Neuro Sciences
PICF: participant information and consent form
RCT: randomized controlled trial

## Data Availability

Raw footage of the video interviews cannot be entirely deidentified and will not be publicly available. Other data set generated during this study, such as deidentified transcripts and anonymized survey data, RCT data, and data analytics, will be available from the corresponding author upon reasonable request.
