# Supplementary material for: Using Digital Media to Improve Dementia Care in India: Protocol for a Randomized Controlled Trial
Source: JMIR Res Protoc. 2022 Jun 2;11(6):e38456. doi: 10.2196/38456 (PMC9204579; doi:10.2196/38456)
Supplement: Multimedia Appendix 1 [file resprot_v11i6e38456_app1.pdf]

alzheimer's association

Applicant: Brijnath, Bianca
Title: Moving Pictures: Using digital media to improve dementia care in India
Program: Alzheimer's Association Research Grant (AARG)
Institution: National Ageing Research Institute
App #: 681419

View Review Information

Close Window Print

Committee: 2020 January Alzheimer's Association Research Grant Review Committee

Scoring range for this committee: 1 Exceptional (Best) - 9 Poor (Worst)

Reviewer Role: Reviewer (1083)

Critique Score: 1 Exceptional

Reviewer Summary: This project can lead to vast improvement in making accurate, relevant, useful, culturally appropriate information easily accessible to informal caregivers of a person with dementia. Persons with dementia and the general public can also benefit from these videos. Making information handy and readily available to caregivers can greatly improve home based care to persons with dementia and lead to improved well being of care receiver and care giver. The tight study design and excellence of the study team maximizes potential for achieving project objectives and major goal while also laying foundations for future research.

A. Significance **Question Score:** 1 Exceptional

A. Significance of the Question Being Studied..

(Applicants appreciate feedback. Please provide a brief paragraph as justification for each score). (Applicants appreciate feedback. Please provide a brief paragraph as justification for each score).:

The significance of the proposed pilot study is the focus on digital media to make accurate and culturally relevant education on dementia and dementia care giving immediately accessible to informal caregivers of persons with dementia living in the home setting. If found to be successful, the use of digital media and smart phone access can lead to a revolution in the way informal caregivers can access accurate, supportive information, find answers to questions on dementia and caregiving, gain support when faced with caregiving challenges, with the potential to lead to better care to persons receiving care in the home and decreased stress for their care givers.

B. Applicant **Question Score:** 2 Outstanding

B. Applicant Information.

(Applicants appreciate feedback. Please provide a brief paragraph as justification for each score). :

The applicant has a strong record of successful research projects and publication in peer reviewed professional journals. She has the knowledge, skills and experience to successfully manage and complete project objectives and primary goal.

C. Work Plan **Question Score:** 1 Exceptional

C. Quality of the Work Plan.
(Applicants appreciate feedback. Please provide a brief paragraph as justification for each score). :
This ambitious, 3 year long project is complex, well-developed and tightly designed to achieve stated objectives and project goal.

An important voice is missing in the study sample identified for participation in qualitative interviews. The story of dementia and dementia care cannot be told without the voices of those who have dementia, who experience receiving personal care from a family member or stranger and who may be influenced by effects of cultural beliefs and norms about dementia. Few informal or formal caregivers ask or consider how persons with dementia experience the loss of cognition and memory, becoming a care receiver or comfort level in needing to ask for what they want or need. Adding the voices of care receivers can contribute valuable information and insight to videos on dementia and dementia caregiving.

Mobility and safety are two critical elements in maintaining the functional and cognitive capacity of a person with dementia especially if the person is an older adult. It will be important for mobility and safety to be discussed in caregiver interviews and considered for subsequent inclusion in the proposed videos.

D. Resources/Budget **Question Score:** 1 Exceptional
D. Quality and Adequacy of Available Resources and Budget..
(Applicants appreciate feedback. Please provide a brief paragraph as justification for each score). :
Resources available to implement this pilot study are extensive and comprehensive involving international multidisciplinary professionals, agencies, and organizations.
The project team is an exceptional resource with each member bringing a high level of experience and expertise to the project. Individual resumes are comprehensive documenting professional background and expertise with special attention to relevance to current project. A major strength is the experience multiple project members bring from a similar project conducted in Australia.

The resource budget requests support dollars within the allotted grant budget with extensive supplemental and in kind supports in personnel and equipment budgets, significantly expanding available resources. The final budget is sufficient to meet project goals.

E. Impact-Risk **Question Score:** 1 Exceptional
E. The Alzheimer's Association is interested in funding investigations that will have a major impact on the field of Alzheimer's and related diseases, including projects that may have an element of risk. However, such projects MUST contain the potential to fuel significant gains and/or insights. Based on this assertion, if this project were successful, could it have a major impact on advancing the field of dementia research? Please select the score which best reflects your determination..
(Applicants appreciate feedback. Please provide a brief paragraph as justification for each score).:
There is minimal risk to study participants.

Success of this project will bring about significant changes, nationally and internationally, in the way people caring for those with dementia, those with dementia and the general public access timely, accurate information on dementia. Study results will generate increased attention to ensuring information is culturally relevant.

Reviewer Role: Reviewer (1457)

Critique Score: 4 Very Good
Reviewer Summary: This is a solid proposal. The workplace is outlined well with outcomes and deliverables. Applicant is well qualified and supported by solid team. Not sure approach is best for the population for most impact.

A. Significance **Question Score:** 4 Very Good

A. Significance of the Question Being Studied..

(Applicants appreciate feedback. Please provide a brief paragraph as justification for each score). (Applicants appreciate feedback. Please provide a brief paragraph as justification for each score).:

Question is significant however would like to have seen more about why moving pictures is the best way to impact this audience and lead to outcomes studied.

B. Applicant **Question Score:** 4 Very Good

B. Applicant Information.

(Applicants appreciate feedback. Please provide a brief paragraph as justification for each score). :

Seems appropriate for the the research outlined and has team in place to support the study. Previous experience and publications support the outlined work.

C. Work Plan **Question Score:** 4 Very Good

C. Quality of the Work Plan.

(Applicants appreciate feedback. Please provide a brief paragraph as justification for each score). :

Workplan is well outlined with outcomes and deliverables. Valid and reliable measures outlined. Supportive interdiscplinary team in place to accomplish work.

D. Resources/Budget **Question Score:** 3 Excellent

D. Quality and Adequacy of Available Resources and Budget..

(Applicants appreciate feedback. Please provide a brief paragraph as justification for each score). :

The budget and resources are appropriate for the work outlined in the proposal. Additional team members seem appropriate to assist with the proposed work.

E. Impact-Risk **Question Score:** 4 Very Good

E. The Alzheimer's Association is interested in funding investigations that will have a major impact on the field of Alzheimer's and related diseases, including projects that may have an element of risk. However, such projects MUST contain the potential to fuel significant gains and/or insights. Based on this assertion, if this project were successful, could it have a major impact on advancing the field of dementia research? Please select the score which best reflects your determination..

(Applicants appreciate feedback. Please provide a brief paragraph as justification for each score).:

Seems innovative as it leverages India’s growing digital presence – 340.2 million Indians are smartphone users, estimated to grow by 84% – to promote an intervention that is accessible to those who have low literacy, live in rural areas, and/or are time poor.

Reviewer Role: Reviewer (365049)

Critique Score: 2 Outstanding

Reviewer Summary: Overall, the research team is highly experienced with a strong history of collaboration and publications. Prior experience heading the Australia Moving Pictures is a huge advantage to the proposed study for several reasons described above. The question is significant - improving dementia care at home - and the means of dissemination (digital media) is highly appropriate (accessible, cost-effective) for this population. The deliverable is highly adaptable for other communities and will be a valuable model for other media-based interventions to improve dementia home care in culturally relevant ways.

A. Significance **Question Score:** 2 Outstanding

A. Significance of the Question Being Studied..

(Applicants appreciate feedback. Please provide a brief paragraph as justification for each score). (Applicants appreciate feedback. Please provide a brief paragraph as justification for each score).:

The applicant aims to produce 9 short films accessible via smartphone and that focus on 9 domains related to dementia

symptoms, clinical care experience, and practical caregiver information to help improve family-based dementia care in India. Up to 50 interviews from health providers (25) and caregivers (25) inform the culturally relevant digital media storyboards. Storyboards are member-checked, and resulting videos are community-evaluated using surveys for feasibility and acceptability.

The problem the applicant addresses is significant because India has the world's highest growing dementia rate and, coupled with a high estimate (90%) of people diagnosed with dementia are unaware of their condition. The use of smartphones to access videos is appropriate, given the high rate of current and projected smartphone users (84%) in 2020, and its lower cost compared to television and print media. Applicant understands value of establishing a firm cultural understanding, rather than assumption, for improved stakeholder and target audience buy-in, acceptance, and impact. Caregiver learning is critical for quality dementia care.

B. Applicant **Question Score:** 1 Exceptional

B. Applicant Information.

(Applicants appreciate feedback. Please provide a brief paragraph as justification for each score). :

The team demonstrates prior collaboration, including co-publishing and clinical training in dementia and digital media work. Two PIs have expertise in dementia care and multicultural communities, and another PI has expertise in cross-cultural mental health and healthcare - understanding methodologies to investigate cultural differences across the region will be critical for producing culturally relevant videos, for potentially greater impact. PI expertise also in communications and technology, digital dissemination, storyboarding, all essential for the proposed work. Further, team will have assistance from the Moving Pictures Australia team. Overall, the team's expertise seems well-rounded and appropriate to successfully implement the study's culturally sensitive digital media aims regarding dementia care. Ellis has several publications on disability media work. Antoniades recently received her PhD in 2017 - her prior experience on the Moving Pictures project will be a great asset, as well as other team members' history of collaboration with the Australia Moving Pictures project. The team includes a current director of the Australian National Ageing Research Institute, with over a decade of experience and publications in dementia health literacy, dementia community care and caregiver understanding of dementia symptoms and more; a research director with over a decade of research in adult home care, non-pharmacological interventions, dementia, and more; Kent and Ellis have an extensive history of co-authorship in digital media studies and disabilities; Loganathan has a decade of work covering dementia and mental health in India, including an online training for caregivers which will be valuable experience for the present proposed caregiver-focused digital media study. Varghese is Head of Geriatric Psychiatry at his institution in India, with over 30 years experience in Alzheimer's and mild cognitive impairment, pathological aging in India. Brijnath has a background in Anthropology and public health, which will lend a critical lens to the cultural piece of this study - relevance, uptake, response, etc. She has published on the culture of dementia care in India, and is PI of Moving Pictures Australia. Her cultural expertise is a critical factor in the Moving Pictures Australia's adaptability to India. This multidisciplinary team has broad expertise in a number of areas appropriate to the proposed study - dementia care, caregiver learning, technology-based interventions and dissemination, anthropology.

C. Work Plan **Question Score:** 2 Outstanding

C. Quality of the Work Plan.

(Applicants appreciate feedback. Please provide a brief paragraph as justification for each score). :

This study follows a validated design - Moving Pictures Australia. Involving participants in the production process has precedence in the Australian Moving Pictures study, so there should be good baseline understanding among the research team of anticipated barriers to circumvent for more sustained and successful involvement and interaction with study participants. Applicant understands value of establishing a firm cultural understanding, rather than assumption, for improved stakeholder and target audience buy-in, acceptance, and impact. The areas of focus are appropriate, practical, and important for developing quality home care: Dementia, help-seeking pathways, the importance of self-care, useful questions to ask health professionals, and how to care for a person with dementia at home on a daily basis. The study follows an

established methodology (UK Medical Research Council guidance for the development and evaluation of complex interventions), which applicant's Australian partners have used prior in the Moving Pictures Study. Applicant noted that efforts will be made for sociological diverse sample of carer interviewees, but that most are women and of middle and

upper class. Having a clearer outline of how researchers will ensure that low-income family carers are included in their study would strengthen the sample diversity, and may improve the cultural relevance of the digital media the research team aims to produce, as low-income families are likely to have less education and financial ability to provide quality care for those living with dementia, and may thus benefit more by having access to digital media. However, some explanation of how many low-income families have access to digital media would be helpful, or how the research team plans to ensure dissemination to populations with decreased access would be helpful.
Similarly, a clearer explanation of the range of sociological diversity of carers at the place of recruitment (NIMHANS) would be helpful.

In Year 1 (objective 1), up to 50 Interviews will be done by two research team members. It is unclear how many team members, if more than the two interviewers, will be conducting analysis. Would be ideal if team members who did not conduct interviews were conducting analysis, or to have the interviewers analyze those interviews they did not conduct. This may be tight timeline to recruit, interview, and analyze qualitative data of up to 50 interviews.
In Year 2 (objective 2), storyboarding will take place, meaning that all interviews and analysis must be completed. Would be good to know if this same rapid timeline was successful in the Moving Pictures Study.

The nominal group technique for cultural appropriateness and critical review of storyboard will include a wide range of people for a good, thorough review process. Subsequent member checking by interviewees will be critical, and the applicant has proposed this, showing their commitment to center the carer perspective that will, as stated before, likely have significant influence on how well digital media is received, shared, and understood, potentially for better care and care-seeking.
Reserving a portion of evaluation surveys to be done in person would may help ensure a more diverse range of socio-economic sample. For older carers, online and even telephone interviews may be less comfortable or accessible (online).
No argument was made for why eligible community members had to be literate, as digital media is a great way to educate those who are illiterate, and illiterate people also use smartphones.
Year 2/3 (Objective 3) will be to evaluate the impact of these resources on care and carer burden.
Primary outcomes are feasibility and acceptability of a full RCT.
40 participants in intervention arm, and 20 controls – receive care and access to digital media; receive care and access to healthy living info (non-dementia) - this seems appropriate for this study at this stage of development.

The applicant provided good argument for ability to recruit caregivers in a short period of time based on prior study experience.
The project offers nine videos that are culturally-specific, scalable, and cost-effective. There is careful attention paid to understanding caregiver perspectives and knowledge gaps regarding dementia care. This study seems highly transferrable to other communities and cultures.

D. Resources/Budget **Question Score:** 2 Outstanding
D. Quality and Adequacy of Available Resources and Budget..
(Applicants appreciate feedback. Please provide a brief paragraph as justification for each score). :
The budget seems appropriate for the proposed activities and travel, particularly with the additional NARI donation of $5000 for travel. Resources seem adequate - recruitment will be greatly facilitated by the 50,000 dollars of clinical staff to assist with interview and pilot recruitment. The applicant has thorough knowledge of financial requirements to successfully carry out a study given prior experience on the Australian Moving Pictures study.

E. Impact-Risk **Question Score:** 2 Outstanding
E. The Alzheimer's Association is interested in funding investigations that will have a major impact on the field of Alzheimer's and related diseases, including projects that may have an element of risk. However, such projects MUST contain the potential to fuel significant gains and/or insights. Based on this assertion, if this project were successful, could it have a major impact on advancing the field of dementia research? Please select the score which best reflects

your determination..

(Applicants appreciate feedback. Please provide a brief paragraph as justification for each score).:

The potential impact is significant for dementia care in India. Digital media is highly accessible, and caregivers are in need of well-designed, informative, and culturally-specific resources to improve their understanding of dementia, resources, practical guidance, self-care, and seeking clinical care. These videos will be the product of a validated methodology that has centralized the carer perspective and culture for potentially quicker and more widespread uptake and greater impact. Risk seems minimal.
